# Pressure Drop of Microchannel Plate Fin Heat Sinks

**DOI:** 10.3390/mi10020080

**Published:** 2019-01-24

**Authors:** Zhipeng Duan, Hao Ma, Boshu He, Liangbin Su, Xin Zhang

**Affiliations:** 1 School of Mechanical, Electronic and Control Engineering, Beijing Jiaotong University, Beijing 100044, China; hebs@bjtu.edu.cn (B.H.); 16116368@bjtu.edu.cn (L.S.); zhangxin@bjtu.edu.cn (X.Z.); 2 Beijing Key Laboratory of Powertrain for New Energy Vehicle, Beijing Jiaotong University, Beijing 100044, China

**Keywords:** pressure drop, microchannels, heat sinks, slip flow, electronic cooling

## Abstract

The entrance region constitutes a considerable fraction of the channel length in miniaturized devices. Laminar slip flow in microchannel plate fin heat sinks under hydrodynamically developing conditions is investigated semi-analytically and numerically in this paper. The semi-analytical model for the pressure drop of microchannel plate fin heat sinks is obtained by solving the momentum equation with the first-order velocity slip boundary conditions at the channel walls. The simple pressure drop model utilizes fundamental solutions from fluid dynamics to predict its constitutive components. The accuracy of the model is examined using computational fluid dynamics (CFD) simulations and the experimental and numerical data available in the literature. The model can be applied to either apparent liquid slip over hydrophobic and superhydrophobic surfaces or gas slip flow in microchannel heat sinks. The developed model has an accuracy of 92 percent for slip flow in microchannel plate fin heat sinks. The developed model may be used to predict the pressure drop of slip flow in microchannel plate fin heat sinks for minimizing the effort and expense of experiments, especially in the design and optimization of microchannel plate fin heat sinks.

## 1. Introduction

Fluid flow in microchannels has emerged as an important research area. This has been motivated by their various applications such as medical and biomedical use, computer chips, and chemical separations. The high level of heat dissipation requires a dramatic reduction in the channel dimensions. The high flux heat dissipation from high-speed microprocessors provided the impetus for studies on heat transfer in microchannels [1]. It is well known that the greatest challenge is overheating because of an increasing power flux and a higher thermal resistance in computer chips [2]. Due to a rapid increase in power density and miniaturization, very high heat flux chip cooling requires a large flow rate, which can lead to a significant pressure drop. The reliability, performance, and power dissipation of interconnects and transistors are heavily dependent on the operating temperature [2]. An effective cooling scheme for use in chip technologies needs to be developed to solve cooling limitations. One potential solution to the thermal management of a chip is to use microchannel heat sinks due to the small size and high heat transfer coefficient. Copper heat sinks with integrated microchannels are expected to dominate heat sink applications to handle high heat removal rates. These practical advantages of microchannel heat sinks have stimulated experimental, theoretical, and numerical research [3,4,5,6,7,8,9,10,11,12,13,14,15,16,17,18,19,20,21,22,23,24,25,26]. The use of microchannel heat sinks is becoming more common in various industrial applications. The flow and heat transfer in microchannel/nanochannel heat sinks have become subjects of growing research attention, and the analysis of microchannel heat sinks has become increasingly important.

Microchannels are the fundamental part of microfluidic systems. In addition to connecting different devices, microchannels are also utilized as biochemical reaction chambers, in physical particle separation, in inkjet print heads, in infrared detectors, in diode lasers, in miniature gas chromatographs, in aerospace technology, or as heat exchangers for cooling computer chips. Understanding the flow characteristics of microchannel flows is very important in determining the pressure drop, heat transfer, and transport properties of the flow for minimizing the effort and expense of experiments, especially in the design and optimization of microchannel plate fin heat sinks. Microchannel heat sinks have received considerable attention owing to their high surface-area-to-volume ratio, large convective heat transfer coefficient, and small mass and volume. For the effective design and optimization of microchannel heat sinks, it is significant to understand the fundamental characteristics of fluid flow and heat transfer in microchannels.

The purpose of this paper is to study the pressure drop characteristics of fluid flow through microchannel plate fin heat sinks. The developing slip flow friction factor Reynolds number parameter is determined for the laminar regime. Following this, there we analyze the pressure drop through microchannel plate fin heat sinks.

In this study, the developing laminar flow is analyzed through plate fin heat sinks with velocity slip boundary conditions, and semi-analytical closed-form solutions are obtained for the friction factor and Reynolds number product and pressure drop in terms of the channel aspect ratio, channel length, hydraulic diameter, Reynolds number, and modified Knudsen number. The primary goal of the present paper is to provide a general simple pressure drop model of fluid flow through microchannel plate fin heat sinks.

## 2. Literature Review

The Knudsen number (*Kn*) relates the molecular mean free path of gas to a characteristic dimension of the duct. The Knudsen number is very small for continuum flows. However, for microscale gas flows, the gas mean free path becomes comparable to the characteristic dimension of the duct. Rarefaction effects must be considered in gases in which the molecular mean free path is comparable to the channel’s characteristic dimension. The continuum assumption is no longer valid, and the gas exhibits non-continuum effects such as velocity slip and temperature jump at the channel walls. Traditional examples of non-continuum gas flows in channels include low-density applications such as high-altitude aircraft or vacuum technology. The recent development of microscale fluid systems has motivated great interest in this field of study. Microfluidic systems should take into account slip effects. There is strong evidence to support the use of Navier–Stokes and energy equations to model the slip flow problem, while the boundary conditions are modified by including velocity slip and temperature jump at the channel walls. The slip length can be interpreted as the distance from the wall that the slip velocity profile extends to if extrapolated away from the boundary. In gas flows, the slip length is related to the Knudsen number, while in liquid flows, the slip length depends on the surface microstructure [27,28,29,30,31,32,33,34,35,36,37]. When considering liquids, the molecular mean free path may be replaced by the slip length and the Knudsen number may be replaced by the modified Knudsen number. The slip lengths reported experimentally span several orders of magnitude, from molecular lengths up to hundreds of nanometers with dependence on wetting conditions, surface roughness structure (shape and distribution), dissolved gas, surface charge, shear rate, and pressure. Hydrophobic coatings on the walls of the microchannels facilitate larger flow rates compared to hydrophilic counterparts for the same pressure drop as they offer less resistance to flow. Even though there have been considerable efforts made to study the fluid transport in hydrophobic microchannels in the fully developed regime, not much attention had been paid to the entrance effects. The entry region is not yet so deeply investigated. There is a clear need to investigate the coupled characteristics between velocity slip and entrance effects in order to understand microchannel flows.

There is extensive literature appearing on micro-scale slip flow and heat transfer. Morini et al. [38] investigated slip flow in rectangular microchannels. They presented the 2D velocity distribution of steady-state, laminar slip flow for Newtonian fluids in a hydrodynamically fully developed region. Morini et al. [39] focused on the role of the main scaling effects in adiabatic and diabatic microchannels, and analyzed the effects of viscous dissipation, conjugate heat transfer, and entrance effects on the mean value of the Nusselt number.

Wang [40] developed accurate analytical solutions for fully developed slip flow and H1 heat transfer in rectangular and equilateral triangular ducts. He pointed out that both velocity slip and temperature jump have significant influences on the Poiseuille and Nusselt numbers. Further, Wang [41] used an efficient analytical method to investigate H1 and H2 forced convection heat transfer in rectangular ducts, especially for large aspect ratios.

Some studies on the developing region in microchannels have been presented [42,43,44,45,46,47,48,49,50,51,52,53,54]. Steinke and Kandlikar [42] identified single-phase heat transfer enhancement techniques for use in microchannels. They speculated that this increase in heat transfer performance from these techniques could place a single-phase liquid system in competition with a two-phase system, thus simplifying the overall complexity and improving the overall reliability. However, they pointed out that the added pressure drop resulting from the techniques should be carefully evaluated. Steinke and Kandlikar [43] reviewed the available literature on single-phase liquid friction factors in microchannels and indicated that there seems to be a common thread between all of the papers that had reported some form of discrepancy between the experimental data and the predicted theoretical values. Those authors that have not discussed the developing length seem to be the same authors reporting the discrepancies. Further, the authors that have considered the added friction factor for the developing region report good agreement with the predicted traditional theory.

Ranjith et al. [44] studied numerically the hydrodynamics of a steady developing flow between two infinite parallel plates with hydrophilic and hydrophobic surfaces using dissipative particle dynamics. The hydrophobic and hydrophilic surfaces were modeled using partial-slip and no-slip boundary conditions, respectively. The simulation results of the developing flow are in good agreement with analytical solutions from Duan and Muzychka [45] for no-slip and partial-slip surfaces. Mishan et al. [46] experimentally investigated developing flow and heat transfer in rectangular microchannels. The experimental results of pressure drop confirmed that the data presented by other research can be due to entrance effects.

Kohl et al. [47] experimentally investigated entrance effects and hydrodynamic developing flow for pressure drop calculations. The experimental results suggested that it is important to include entrance effects, especially for the channels with $L/D_{h}$ < 300.

Bayraktar and Pidugu [48] indicated that even though a tremendous effort in microfluidics research is currently underway, there is little work done to study the entry flow in microchannels. Researchers have generally assumed microchannel flows to be laminar and fully developed, ignoring entrance effects. However, in the entry region, the velocity distribution and skin friction show significant variations in the stream-wise direction, which could influence separation efficiency in processes such as electrophoresis.

Barber and Emerson [49] conducted a numerical investigation of gaseous slip flow at the entrance of circular and parallel plate microchannels using a two-dimensional Navier–Stokes solver. Barber and Emerson [49] examined the role of Reynolds and Knudsen numbers on the hydrodynamic development length at the entrance to circular and parallel plate microchannels. They carried out numerical simulations over a range of Knudsen numbers covering the continuum and slip flow regimes. They proposed expressions for the hydrodynamic entrance length but did not provide any expressions for pressure drop.

Vocale and Spiga [50] conducted a numerical study of hydrodynamically developing flow in the entrance region of rectangular microchannels with different values of aspect ratio using a solver based on the finite element method with first-order slip boundary conditions.

Hettiarachchi et al. [51] numerically studied three-dimensional laminar slip flow and heat transfer in rectangular microchannels having constant-temperature walls using the finite-volume method for thermally and simultaneously developing flows. They evaluated the effect of rarefaction on the hydrodynamically developing flow field, pressure gradient, and entrance length.

Recently, many efforts have been made applying innovative geometries [3,4,5,6,7,8,9,10,11,12,13], using fluids with excellent thermal features [14,15,16,17], and utilizing micro-pin-fins [15,16,17,18,19,20,21] in order to enhance the performance of microchannel heat sinks. 

Bahiraei and Heshmatian [5] evaluated the flow, heat transfer, and second law characteristics of a hybrid nanofluid containing grapheme–silver nanoparticles inside two new microchannel heat sinks. 

Khan et al. [6] numerically investigated the thermal and hydraulic performances of various geometric shapes of a microchannel heat sink with water as the cooling fluid. The performances of seven microchannel shapes were compared at the same microchannel hydraulic diameter and the same average height of the bottom silicon substrate. Their results showed that an inverse trapezoidal shape gives the lowest thermal resistance for a Reynolds number up to 300, and the values of friction factor and Reynolds number product are almost similar for all the shapes because of the constant hydraulic diameter. Ansari and Kim [7] proposed a transverse-flow arrangement concept of a double-layer microchannel heat sink. They performed a numerical analysis using three-dimensional Navier–Stokes equations for the entire heat sink domain to evaluate the thermal and hydraulic performance of the proposed heat sink. 

Al Siyabi et al. [8] experimentally examined the behavior of a multilayered microchannel heat sink integrated with a concentrating photovoltaic assembly. In their work, experiments were conducted to evaluate the thermal performance of multilayered microchannel heat sinks for different numbers of layers, different heat transfer fluid flow rates, and different heating power rates. They further developed a numerical model to analyze the heat sink behavior for measurements that cannot be obtained using the experimental approach.

Shen et al. [9] proposed a modification of double-layer microchannel heat sinks into a wavy configuration with a swap of the upper and lower layer design. They demonstrated that this design could effectively improve the comparatively low cooling effectiveness at the upper layer, yet the swap of flow could promote re-developing of the flow field and heat transfer, accordingly. Some studies on flow and heat transfer characteristics in double-layered microchannel heat sinks have also been presented [10,11,12,13].

Xia et al. [14] provided an overall analysis of nanofluids flowing through microchannel heat sinks. They obtained the temperature distribution on the substrate of microchannel heat sinks. Their results indicated that the thermal conductivity and dynamic viscosity of Al_2_O_3_ and TiO_2_ nanofluids are both improved with increasing particle volume fraction. The thermal motion of nanoparticles could promote the interruption of laminar flow and intensify the heat transfer between fluids and channel walls.

Hassani et al. [15] investigated the effects of different interruptions of fins on the transport characteristics of a nanofluid-cooled electronic heat sink with a chevron shape. Seven interruptions of fins were studied, and water-based nanofluid with Al_2_O_3_ nanoparticles at volume fractions of 0.5% and 1.0% were tested as the coolant in a laminar flow regime. 

Zargartalebi and Azaiez [16] analyzed the effects of nanoparticle properties and pin sizes on heat removal performance using a nanofluid two-component model. Their results indicated that the nanoparticle distribution plays an important role in heat transfer. 

Xu and Wu [18] experimentally investigated water flow and heat transfer characteristics in silicon micro-pin-fin heat sinks with various pin-fin configurations and a conventional microchannel. Their results indicated the better heat transfer performance of the micro-pin-fin heat sinks than of the conventional microchannel. The dominant mechanism of heat transfer enhancement caused by the micro-pin-fins is the hydrodynamic effects, including fluid disturbance as well as the breakage and re-initialization of the thermal boundary layer near the wall of the heat sinks.

Ansari and Kim [19] presented a novel hotspot-targeted cooling technique combining microchannels and pin-fins for efficient thermal management of microprocessors with heterogeneous power distributions. The performance of the proposed microchannel-pin-fin hybrid heat sink was evaluated numerically and compared with that of a simple microchannel heat sink in their study. They indicated that the hybrid heat sink exhibited a remarkable improvement in thermal performance compared to the non-hybrid heat sink with a reasonable increase in the pumping power. 

Adewumi et al. [21] numerically investigated steady incompressible flow and forced convection heat transfer through a microchannel heat sink with micro-pin-fin inserts for both fixed and variable axial lengths. The effects of the micro-pin-fins on the optimized microchannel were evaluated in detail.

Ribs mounted in the microchannel heat sink generally result in a higher heat transfer coefficient but are usually accompanied by a higher pressure drop per unit length. Khan et al. [22] presented an analysis of microchannel heat sinks with ribbed channels in various configurations using the three-dimensional Navier–Stokes equations and compared them to smooth channels in a Reynolds number range of 100–500. Their results indicated that the thermal resistance of microchannels was greatly reduced by introducing ribs, and the pressure drop was increased greatly because of the ribs. 

Chai et al. [23] presented a detailed numerical study on local laminar fluid flow and heat transfer characteristics in microchannel heat sinks with tandem triangular ribs for a Reynolds number of 443. Three-dimensional conjugate heat transfer models considering entrance effect, viscous heating, and temperature-dependent thermophysical properties were employed in their studies [23,24]. Their results showed that the triangular ribs could significantly reduce the temperature rise of the heat sink base and efficiently prevent the drop of the local heat transfer coefficient along the flow direction, but also result in a higher local friction factor than the straight microchannel. Further, the effects of the geometry and arrangement of triangular ribs on the thermohydraulic performance were examined by the variations in the average friction factor and Nusselt number for Reynolds numbers ranging from 187 to 715. 

Chai et al. [25] performed a study of the laminar flow and heat transfer characteristics in an interrupted microchannel heat sink with ribs in the transverse microchambers. They investigated five different rib configurations, including rectangular, backward triangular, diamond, forward triangular, and ellipsoidal. The role of such ribs in the velocity contour, pressure distribution and temperature distribution, and the local pressure drop and heat transfer characteristics in such microchannel heat sinks was studied.

Zhai et al. [26] summed up the empirical correlations of laminar convective heat transfer in microchannel heat sinks in previous studies. They established an empirical model of laminar convective heat transfer in microchannel heat sinks. Further, a corresponding experiment and simulation were used to validate the accuracy of their theoretical model.

Fully developed flows have been widely investigated in different geometries, in continuum flow and slip flow conditions. In the case of developing flows with slip, only parallel plates and circular ducts are considered in the literature due to the slip boundary conditions which make this particular hydrodynamically developing flow problem even more complicated. A survey of the available literature indicates a shortage of pressure drop information for three-dimensional entrance flows with velocity slip boundary conditions, such as relatively short plate fin microchannels where the entrance region plays a very important role. There is currently no published model and data for pressure drop which can be utilized by the research community in the design and optimization of microchannel plate fin heat sinks. The entrance region in a microchannel heat sink is particularly of interest due to the presence of comparatively large pressure drop and heat transfer. Wall shear stress effects and velocity distributions vary significantly at the entrance region, and these may eventually affect the separation efficiency of the microfluidic processes. Moreover, the entrance region in hydrophobic channels is much longer than in hydrophilic channels. Reduction of the entrance length is very important for the design of some types of lab-on-a-chip devices. Given that the convective heat transfer behavior in the developing region differs from that in the fully developed region, and given that many microchannel heat sinks are short, this effect of the entrance region is significant. The apparent friction factor and Reynolds number product *f*_app_*Re* could be significantly higher than the fully developed value of friction factor and Reynolds number product *fRe*. The first approximate solution was that for the parallel plates obtained by Sparrow et al. [52] using Targ’s linearization theory. Later, Quarmby [53] extended this technique to a circular tube.

Even though a tremendous effort in microchannel heat sink research is currently underway and a vast amount of literature is available for an interested researcher, there is little work performed to study the entrance flow in microchannel heat sinks. Researchers have generally assumed microchannel heat sink flows to be fully developed, ignoring entrance effects; therefore, it may be pointed out that most of the statements, formulas, and charts are valid only for long ducts. When dealing with fluid flow within microchannels, in most applications, the short length of the channels is not enabling the flow to ignore entrance effects and completely reach the fully developed regime. However, in the entrance region, skin friction shows quite significant variations. The deviation of experimental data from fully developed theoretical predictions could be misinterpreted as an early transition to turbulence or other reasons. Although numerous papers have proposed reasons for the conflicting results obtained by different researchers, it can be still concluded that no universally accepted physical interpretation has been found among studies focusing on the characterization of friction factor/pressure drop for microchannel flows. A survey of the available literature indicates a shortage of information for microchannel heat sink entrance flows in the slip regime, such as short plate fin microchannel heat sinks. There is currently no published model or tabulated data for pressure drop which can be utilized by the research community. The lack of a general pressure drop model of fluid flow through microchannel plate fin heat sinks is a major problem. This paper is concentrated on simple compact modeling methods for predicting microchannel plate fin heat sink pressure drop.

## 3. Theoretical Analysis

**Figure 1:**
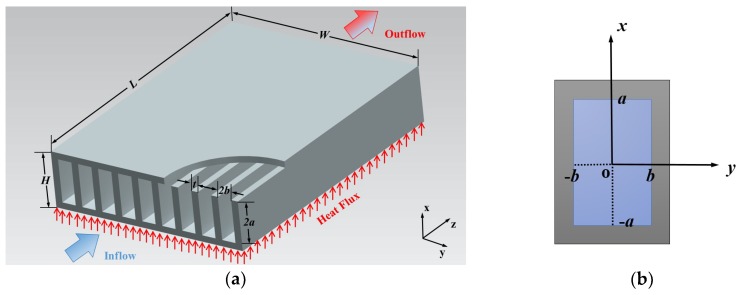
Schematics of the microchannel plate fin heat sink. (**a**) Microchannel plate fin heat sink; (**b**) Computational unit.

The geometry of a microchannel heat sink is shown in Figure 1a. The length of the heat sink is *L*, the width is *W*, and the height is *H*. The top surface is insulated, and the bottom surface is uniformly heated. A coolant passes through a number of microchannels along the *z* axis and takes heat away from the heat-dissipating electronic component attached below. The flow in the channels is steady, laminar, and developing. There are *N* channels, and each channel has a height 2*a* and width 2*b*. The thickness of each fin is *t*. At the channel wall, the slip flow velocity boundary condition is applied to calculate the apparent friction factor and Reynolds number product and pressure drop. One of the most fundamental problems in fluid dynamics is that of laminar flow in circular and non-circular channels under a constant pressure gradient. The starting point in the theoretical discussion will be the definition of the friction factor and Reynolds number parameter. Upon obtaining the velocity distribution *u*(*x*, *y*) and mean velocity $\bar{u}$, the friction factor and Reynolds number parameter may be defined using the simple expression denoted in some texts by the Poiseuille number:

(1) $$Po_{D_{h}}=\frac{\bar{\tau }\;D_{h}}{\mu \;\bar{u}}=\frac{\left(-\frac{A}{P}\frac{dp}{dz}\right)D_{h}}{\mu \;\bar{u}}=\frac{fRe_{D_{h}}}{2}$$

The above grouping *Po* is interpreted as the fully developed dimensionless average wall shear. The fully developed mean wall shear stress may also be related to the pressure gradient by means of the force balance $\bar{\tau }=-A/Pdp/dz$.

We examine the momentum equation and consider the various force balances using the method of scale analysis. Comparing the force scale between friction and inertial forces, we obtain the following relation:

(2) $$\frac{\mu \frac{\partial^{2}u}{\partial y^{2}}}{\rho u\frac{\partial u}{\partial x}}\sim \frac{\mu \frac{U}{D_{h}^{2}}}{\frac{\rho U^{2}}{L}}=\frac{L}{D_{h}Re_{D_{h}}}=\xi$$

where *ξ* is the non-dimensional channel length. This analysis demonstrates that inertial forces are quite important for short ducts where *ξ* << 1. The fluid flow behavior in the developing region differs from that in the fully developed region. The parameter $L/\left(D_{h}Re\right)$ is always a significant parameter in internal fluid flows. The flow behaves differently and is dominated by different mechanisms as the parameter $L/\left(D_{h}Re\right)$ changes.

In many practical applications, the length of the channel in the developing region therefore forms a major portion of the flow length through a microchannel. To account for the developing region, the pressure drop equations are presented in terms of an apparent friction factor. The apparent friction factor accounts for the actual pressure drop due to friction and the developing region effects. It represents an actual value of the friction factor over the flow length between the inlet and the location under consideration. Therefore, the apparent friction factor, *f*_app_, must be utilized to calculate the factual pressure drop. The apparent flow friction factor is used in this paper since it incorporates the pressure drops caused both by the wall shear stress due to the significant velocity gradient normal to the wall and by the momentum flux variation due to the change of velocity field from a uniform profile at the inlet to a specific profile downstream in the channel. Researchers have generally assumed microchannel heat sink flows to be fully developed, ignoring entrance effects; therefore, care should be exercised as most of the statements, formulas, and charts are valid only for long ducts. The long duct criterion will be discussed later in this paper.

Generally, there are three main components that contribute to the overall pressure drop. The inlet and exit losses need to be quantified for the microchannel. The hydrodynamic frictional pressure loss needs to be carefully evaluated. Summing all of the frictional and inlet and exit dynamic losses, the total pressure drop model function is given in terms of Bernoulli’s equation,

(3) $$\Delta P=\left[K_{c}+4\left(f_{\mathrm{app}}Re_{D_{h}}\right)\frac{L}{D_{h}Re_{D_{h}}}+K_{e}\right]\frac{1}{2}\rho {\bar{u}}^{2}=\left[K_{c}+4\left(f_{\mathrm{app}}Re_{D_{h}}\right)\xi +K_{e}\right]\frac{1}{2}\rho {\bar{u}}^{2}$$

where *K_c_* and *K_e_* represent the contraction and expansion loss coefficients due to area changes. The friction factor used in Equation (3) is the apparent friction factor and accounts for the developing region. Fluid flow modelling in a plate fin heat sink is essentially a simultaneously developing hydraulic and thermal boundary layer problem in rectangular ducts. The flow may become fully developed if the heat sink channel is sufficiently long in the flow direction or with relatively small fin spacing; however, this is very unlikely for microchannel heat sinks for electronic cooling applications. The hydrodynamically developing flow can become quite important in microchannels. Due to the often-short lengths, the developing flow could dominate the entire flow length of the microchannel. When considering the developing flows, the pressure drop is now related to an apparent friction factor. The apparent friction factor, *f*_app_, for a rectangular channel may be computed using a form of the model developed by Duan and Muzychka [45] for developing laminar flow. Duan and Muzychka [45] demonstrated that the boundary layer behavior in a circular tube entry is substantially identical to that on a flat plate. As the boundary layer develops further downstream, the effects of geometry become gradually more pronounced. We will take advantage of the asymptotic limit for developing an approximate model for predicting pressure drop for plate fin heat sinks. The apparent friction factor consists of two components just for convenience. Actually, the apparent friction factor should be utilized to calculate the pressure drop. The first is the friction factor from the familiar theory for the fully developed flow, and the second is the pressure defect. The first term is the frictional loss resulting from the fully developed flow. The second term represents the added pressure drop due to the developing flow region. It is convenient to report the pressure drop in a developing flow as equal to that for a fully developed flow plus a correction term *G*(*ξ*) representing additional pressure drop which exceeds the fully developed pressure drop. Thus, the difference between the apparent friction factor and Reynolds number product over a length and the fully developed friction factor and Reynolds number product is expressed in terms of an incremental pressure defect *G*(*ξ*):

(4) $$f_{\mathrm{app}}Re=fRe+G\left(\xi \right)$$

The commonly used customary incremental pressure drop number (also denoted in some texts by Hagenbach’s factor) is given by

(5) $$k=\left(f_{\mathrm{app}}-f\right)\frac{4L}{D_{h}}=\left(f_{\mathrm{app}}Re-fRe\right)4\xi =4\xi G\left(\xi \right)$$

The Navier–Stokes equations are assumed to be valid in their traditional form, and wall slip is merely modeled through a modification of the boundary condition. Rectangular geometries are of particular interest in microfluidics applications. We may now examine the solution for rectangular ducts for slip flow. A schematic diagram of the rectangular cross section is showed in Figure 1b. When *ξ* >> 1, the fully developed flow momentum equation in Cartesian coordinates reduces to the form

(6) $$\frac{\partial^{2}u}{\partial x^{2}}+\frac{\partial^{2}u}{\partial y^{2}}=\frac{1}{\mu }\frac{dp}{dz}$$

The velocity distribution must satisfy the slip boundary condition at the walls. The slip boundary condition that is applied in the analysis both of slip gas flows and of liquid flows over superhydrophobic surfaces takes the following form. The local slip velocity is proportional to the local velocity gradient normal to the wall. Due to symmetry, the boundary conditions are

(7) $$u=-\lambda \frac{\partial u}{\partial y}\;at\;y=b,\;0\leq x<a$$

(8) $$u=-\lambda \frac{\partial u}{\partial x}\;at\;x=a,\;0\leq y<b$$

(9) $$\frac{\partial u}{\partial y}=0\;at\;y=0,\;0\leq x\leq a$$

(10) $$\frac{\partial u}{\partial x}=0\;at\;x=0,\;0\leq y\leq b$$

where *λ* generally denotes the slip length for either gases or liquids, which is defined based on the physics of the fluid flow. The slip length can be used to characterize the type of flow in channels: if *λ* = 0, the flow is no-slip flow; if *λ* = ∞, the flow is plug flow; and intermediate values of *λ* represent partial slip flow. For gas slip flow, Maxwell’s first order correction gives

(11) $$u=-\lambda_{p}\frac{2-\sigma }{\sigma }\frac{\partial u}{\partial y}\;at\;y=b,\;0\leq x<a$$

(12) $$u=-\lambda_{p}\frac{2-\sigma }{\sigma }\frac{\partial u}{\partial x}\;at\;x=a,\;0\leq y<b$$

where *λ_p_* is the molecular mean free path. The constant *σ* denotes the tangential momentum accommodation coefficient. Equations (11) and (12) are mathematically equivalent to the first-order correction commonly employed in the analysis of rarefied gas flow in the slip flow regime. It is convenient to define a modified Knudsen number as Kn^*^ = *Kn*(2 − *σ*)/*σ*. A nondimensional number similar to the modified Knudsen number (Kn^*^) can be defined for liquid slip flow, i.e., the ratio of slip length to a characteristic dimension of the flow field, and the presently developed model can then be utilized for liquid flows over superhydrophobic surfaces to predict pressure drop. To look at it from a slightly more general mathematical point of view, when the no-slip condition on the solid surfaces is partially relaxed, the molecular mean free path and the term involving the accommodation coefficient ($\lambda_{p}\left(2-\sigma \right)/\sigma$) and the slip length (λ) have identical mathematical meaning.

Following Ebert and Sparrow [55], using the Method of Eigenfunction Expansions, a solution of the velocity may be assumed as follows:

(13) $$u=\frac{b^{2}}{\mu }\frac{dp}{dz}\sum_{n=1}^{\infty }X_{n}\left(\frac{x}{a}\right)\cos\left(\delta_{n}\frac{y}{b}\right)$$

in which the *δ_n_* are a set of eigenvalues, the *X_n_* are a set of functions of *x/a*, and the cos(*δ_n_*_·_*y*/*b*) are a set of eigenfunctions. This solution satisfies the boundary condition, Equation (9). Furthermore, substituting the velocity solution into the boundary condition, Equation (7), we obtain

(14) $$\delta_{n}\tan\delta_{n}=\frac{b}{\lambda }$$

The characteristic length scale in the present analysis is defined as the hydraulic diameter.

(15) $$Kn_{D_{h}}=\frac{\lambda_{p}}{D_{h}}=\frac{\lambda_{p}}{\frac{4b}{1+\varepsilon }}$$

Thus,

(16) $$\delta_{n}\tan\delta_{n}=\frac{1}{\frac{4}{1+\varepsilon }Kn_{D_{h}}^{*}}$$

The eigenvalues *δ_n_* can be obtained from Equation (16). Finally, the velocity distribution is obtained as follows:

(17) $$u=\frac{b^{2}}{\mu }\frac{dp}{dz}\sum_{n=1}^{\infty }\frac{2\sin\delta_{n}\cos\left(\delta_{n}\frac{y}{b}\right)}{\delta_{n}{}^{2}\left(\delta_{n}+\sin\delta_{n}\cos\delta_{n}\right)}\left[\frac{\cosh\left(\frac{\delta_{n}}{\varepsilon }\frac{x}{a}\right)}{\cosh\left(\frac{\delta_{n}}{\varepsilon }\right)+\frac{4}{1+\varepsilon }Kn_{D_{h}}^{*}\delta_{n}\sinh\left(\frac{\delta_{n}}{\varepsilon }\right)}-1\right]$$

The mean velocity is found by integration of Equation (17) across the section of the duct.

(18) $$\begin{array}{ll} \bar{u} & =\frac{1}{A}\int udA=\int_{0}^{1}\int_{0}^{1}u\;d\frac{x}{a}\;d\frac{y}{b}\\ & =\frac{b^{2}}{\mu }\frac{dp}{dz}\sum_{n=1}^{\infty }\frac{2\varepsilon \sin^{2}\delta_{n}}{\delta_{n}{}^{4}\left(\delta_{n}+\sin\delta_{n}\cos\delta_{n}\right)}\left[\frac{\sinh\left(\frac{\delta_{n}}{\varepsilon }\right)}{\cosh\left(\frac{\delta_{n}}{\varepsilon }\right)+\frac{4}{1+\varepsilon }Kn_{D_{h}}^{*}\delta_{n}\sinh\left(\frac{\delta_{n}}{\varepsilon }\right)}-\frac{\delta_{n}}{\varepsilon }\right] \end{array}$$

We can obtain the friction factor and Reynolds number product from the above equations in terms of the aspect ratio and the slip coefficient.

(19) $$\begin{array}{ll} fRe_{D_{h}} & =\frac{2\left(-\frac{A}{P}\frac{dp}{dz}\right)D_{h}}{\mu \bar{u}}\\ & =\frac{4}{{\left(1+\varepsilon \right)}^{2}\sum_{n=1}^{\infty }\frac{\varepsilon \sin^{2}\delta_{n}}{\delta_{n}{}^{4}\left(\delta_{n}+\sin\delta_{n}\cos\delta_{n}\right)}\left[\frac{\delta_{n}}{\varepsilon }-\frac{\sinh\left(\frac{\delta_{n}}{\varepsilon }\right)}{\cosh\left(\frac{\delta_{n}}{\varepsilon }\right)+\frac{4}{1+\varepsilon }Kn_{D_{h}}^{*}\delta_{n}\sinh\left(\frac{\delta_{n}}{\varepsilon }\right)}\right]} \end{array}$$

The limit of Equation (19) corresponds to a parallel-plate channel for *ε*→0:

(20) $$f{Re}_{D_{h}}=\frac{24}{1+12Kn_{D_{h}}^{*}}$$

It can also be demonstrated that Equation (19) reduces to its no-slip flow limits as Kn^*^→0. The relationship between the flow friction coefficient, *f*, and Reynolds number, *Re*, for a fully developed laminar flow regime in a rectangular channel is only a function of the aspect ratio and may be calculated as follows:

(21) $${\left(fRe_{D_{h}}\right)}_{ns}=\frac{24}{{\left(1+\varepsilon \right)}^{2}\left[1-6\sum_{n=1}^{\infty }\frac{\varepsilon }{\delta_{n}{}^{5}}\tanh\left(\frac{\delta_{n}}{\varepsilon }\right)\right]}$$

Examination of the single-term solution reveals that the single-term approximation is accurate enough for engineering applications. The largest difference occurs when *ε* = 1, which gives an *fRe* value 0.7 percent below the exact value. When greater accuracy is desired, two terms are absolutely enough due to very rapid convergence, and the largest error is less than 0.05%. Considering only the two terms of the exact series, Equation (21) gives

(22) $${\left(fRe_{D_{h}}\right)}_{ns}=\frac{24}{{\left(1+\varepsilon \right)}^{2}\left[1-\frac{192\varepsilon }{\pi^{5}}\left(\tanh\left(\frac{\pi }{2\varepsilon }\right)+\frac{1}{243}\tanh\left(\frac{3\pi }{2\varepsilon }\right)\right)\right]}$$

This equation is founded on theory and is more accurate compared to those obtained by curve fitting [56].

The slip flow friction factor results can be presented conveniently in terms of the normalized Poiseuille number. The Poiseuille number reduction depends on the geometry of the cross section. It is convenient that the Poiseuille number results are expressible to good accuracy by the relation

(23) $$\frac{Po}{Po_{ns}}=\frac{fRe}{{\left(fRe\right)}_{ns}}=\frac{1}{1+\alpha Kn_{D_{h}}^{*}}$$

in which *α* depends on the duct geometry.

For rectangular ducts, the constants *α* are derived from a least-square fit of the Poiseuille number results. The constants *α* are a weak function of the aspect ratio, and the data points are fitted to a simple correlation [57]:

(24) $$\alpha =11.97-10.59\varepsilon +8.49\varepsilon^{2}-2.11\varepsilon^{3}$$

Then, Equation (19) can be simplified to facilitate practical application as follows:

(25) $$fRe=\frac{24}{{\left(1+\varepsilon \right)}^{2}\left[1-\frac{192\varepsilon }{\pi^{5}}\left(\tanh\left(\frac{\pi }{2\varepsilon }\right)+\frac{1}{243}\tanh\left(\frac{3\pi }{2\varepsilon }\right)\right)\right]}\frac{1}{1+\alpha Kn^{*}}$$

It is found that the entrance friction factor and Reynolds number product is of finite value and dependent on Kn^*^ but independent of the cross-sectional geometry [45]. Duan and Muzychka [45] also demonstrated that very near the inlet of circular and parallel plate ducts (*ξ* ≤ 0.001), *f*_app_*Re* is nearly equivalent and independent of duct shape. The boundary layer behavior in the tube entry is substantially identical to that on a flat plate. At the entrance of the duct, the velocity boundary layer starts developing at each wall under the imposed flow acceleration. As long as the thickness of the boundary layer is small compared to the duct dimensions, the boundary layers from different walls do not affect each other appreciably. This explains why very near the inlet of ducts, *f*_app_*Re* is nearly equivalent and independent of duct shape. As the boundary layer develops further downstream (*ξ* > 0.001), the effects of geometry become gradually more pronounced. The incremental pressure defect *G*(*ξ*) for a rectangular channel may be computed using a form of the model developed by Duan and Muzychka [45] for developing laminar flow:

(26) $$G\left(\xi \right)=\frac{1}{3\xi {\left(1+8Kn^{*}\right)}^{2}}-2\sum_{i=1}^{\infty }\frac{\left(3-\exp\left(-4\alpha_{i}{}^{2}\xi \right)\right)\exp\left(-4\alpha_{i}{}^{2}\xi \right)}{\alpha_{i}{}^{2}\xi \left(1+8Kn^{*}+4{\left(\alpha_{i}Kn^{*}\right)}^{2}\right)}$$

Equation (26) is nearly independent of the duct shape and may be used to calculate the friction factor and Reynolds number product for the short asymptote of rectangular ducts for slip flow. We will take advantage of the asymptotic limit for developing an approximate model for predicting pressure drop for plate fin heat sinks. The eigenvalue $\alpha_{i}$ satisfies the following equation [45]:

(27) $$\alpha_{i}J_{0}\left(\alpha_{i}\right)-2\left(1+Kn^{*}\alpha_{i}{}^{2}\right)J_{1}\left(\alpha_{i}\right)=0$$

where $J_{v}\left(x\right)$ is the Bessel function of the first kind of order *ν*. While typical microflows are characterized by *Re <* 100, in a few microfluidic applications such as micro heat exchangers and micromixers, the Reynolds number can reach the order of a few hundreds. It is emphasized that several eigenvalues are sufficiently accurate for all values of *ξ* of engineering interest. The negative exponentials cause rapid convergence, especially if *ξ* is not too small. As an illustration, for a practical engineering application limit of *ξ* ≥ 0.01, only two terms in the summation are really required. It can be demonstrated [45] that the proposed model of Equation (28) strictly correctly approaches the *ξ*→0 asymptote $2/Kn^{*}$ and approaches the *ξ*→∞ asymptote of Equation (19). Therefore, using the simple expression Equation (28), the apparent friction factor and Reynolds number product results can be easily obtained for practical engineering applications without sacrificing much in accuracy.

(28) $$\begin{array}{l} f_{\mathrm{app}}Re=\frac{24}{{\left(1+\varepsilon \right)}^{2}\left[1-\frac{192\varepsilon }{\pi^{5}}\left(\tanh\left(\frac{\pi }{2\varepsilon }\right)+\frac{1}{243}\tanh\left(\frac{3\pi }{2\varepsilon }\right)\right)\right]}\frac{1}{1+\alpha Kn^{*}}\\ \;+\frac{1}{3\xi {\left(1+8Kn^{*}\right)}^{2}}-2\sum_{i=1}^{2}\frac{\left(3-\exp\left(-4\alpha_{i}{}^{2}\xi \right)\right)\exp\left(-4\alpha_{i}{}^{2}\xi \right)}{\alpha_{i}{}^{2}\xi \left(1+8Kn^{*}+4{\left(\alpha_{i}Kn^{*}\right)}^{2}\right)} \end{array}$$

For the inlet and exit pressure losses for a heat sink, Kays and London [58] provide loss coefficients in the form $\Delta P=K\left(\rho {\bar{u}}^{2}/2\right)$ as a function of the ratio of free-flow area to frontal area *φ* = 2*b*/(2*b* + *t*). The graphs of experimental data for laminar flow in Reference [58] have been curve fitted here for laminar flow:

(29) $$K_{c}=0.4\left(1-\varphi^{2}\right)+0.4$$

(30) $$K_{e}={\left(1-\varphi \right)}^{2}-0.4\varphi$$

## 4. Results and Discussion

**Figure 2:**
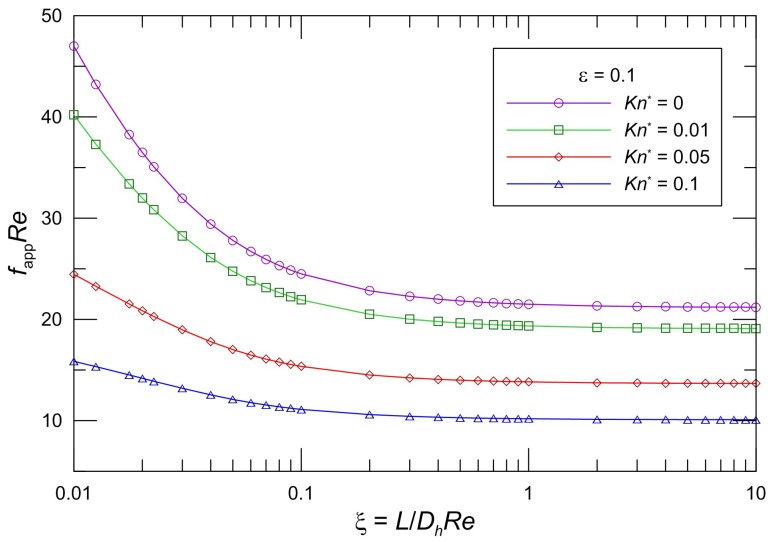
Effect of Kn^*^ on *f*_app_*Re* for rectangular ducts (*ε* = 0.1).

**Figure 3:**
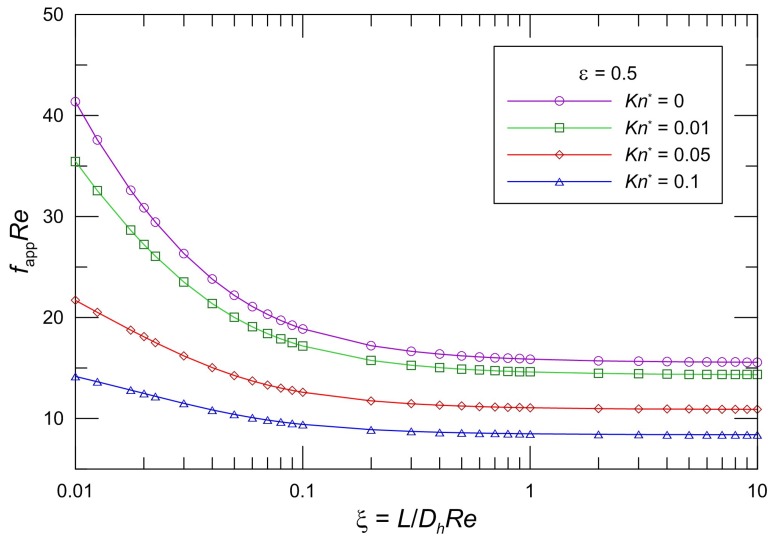
Effect of Kn^*^ on *f*_app_*Re* for rectangular ducts (*ε* = 0.5).

**Figure 4:**
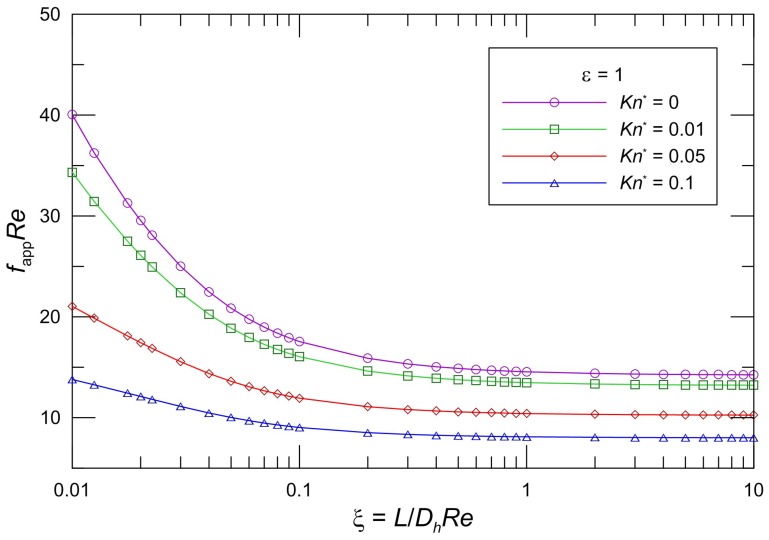
Effect of Kn^*^ on *f*_app_*Re* for rectangular ducts (*ε* = 1).

The developing apparent friction factor and Reynolds number parameter as a function of aspect ratio and modified Knudsen number is illustrated clearly in some graphs. Figure 2, Figure 3 and Figure 4 present the proposed model Equation (28) *f*_app_*Re* in slip flow for various aspect ratios of the rectangular cross section. From an inspection of the graphs, it is seen that *f*_app_*Re* monotonically decreases in the entrance region, and *f*_app_*Re* decreases as the modified Knudsen number increases for the same aspect ratio. The effect of increasing Kn^*^ is to decrease the apparent friction and pressure drop over the channel length very significantly. In addition, the *f*_app_*Re* values decrease with increasing *ε* for the same Kn^*^. Moreover, it is obvious that the pressure gradient for a slip flow is less than that for the corresponding no-slip flow. This effect of the developing region is significant for microchannel plate fin heat sinks.

The values in the entrance region are larger than those in the fully developed region, which demonstrates the critical importance of the entrance region in determining the pressure drop characteristics in microchannel heat sinks. To take into account the entrance effects on the overall pressure drop in the microchannel region, clearly, the dimensionless developing length *ξ* is the proper parameter. Figure 2, Figure 3 and Figure 4 indicate that the local apparent friction factor and Reynolds number product decreases and approaches the fully developed constant value with increasing dimensionless developing length *ξ*. The effects of the aspect ratios on the pressure drop in the slip flow region are investigated in Figure 2, Figure 3 and Figure 4. It is noted that the pressure drop is higher for lower aspect ratios. The pressure drop in microchannel plate fin heat sinks decreases with an increase in the aspect ratios.

Numerical simulations were conducted using the commercial solver ANSYS Fluent 14.0 (Ansys Inc., Canonsburg, PA, USA) with user-defined functions. A structured mesh composed of rectilinear elements was constructed in a preprocessor to define the flow domains. The governing equations were solved with a commercial implementation of the finite volume method using a pressure-based solver and the SIMPLE algorithm. The slip boundary condition was implemented as a user-defined function in FLUENT. The solution algorithms were considered converged when the convergence criterion was satisfied. Local mesh refinement was performed using FLUENT’s grid adaption utility at the flow inlet.

**Figure 5:**
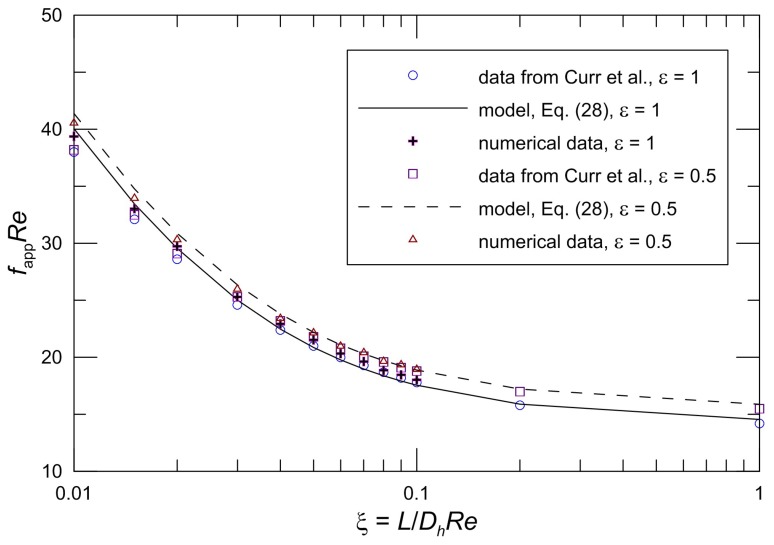
Comparison of *f*_app_*Re* for Curr et al. [59] and new numerical data.

Shah and London [56] presented the results of Curr et al. [59] for hydrodynamically developing laminar flow in rectangular ducts. We validated the model with most of the available developing flow data for the rectangular channel. Figure 5 demonstrates the comparison between the proposed model Equation (28) and the available numerical data from Curr et al. [59] and our new numerical data. The model predictions are in agreement with the numerical solution. It is found that the difference between the model and available data from Curr et al. [59] is less than 7.6%. The maximum difference between our numerical data and the proposed model is less than 3.0%. This result does support the validity of the model. From the figure it is seen that the difference increases with a decrease in the aspect ratio. The difference is smaller and negligible for large aspect ratios. The apparent friction factor and Reynolds number product is significantly higher than the fully developed value. The distribution of *f*_app_*Re* does not depend on the dimensionless hydrodynamic length when the dimensionless hydrodynamic length is approximately unity, which should be the long duct criterion. In the range of *ξ* < 0.06, the *f*_app_*Re* decreases rapidly with increasing *ξ* due to the finite thickness of the boundary layer at the microchannel entrance region. With an increase of *ξ*, the *f*_app_*Re* has a comparatively gently downward tendency.

**Figure 6:**
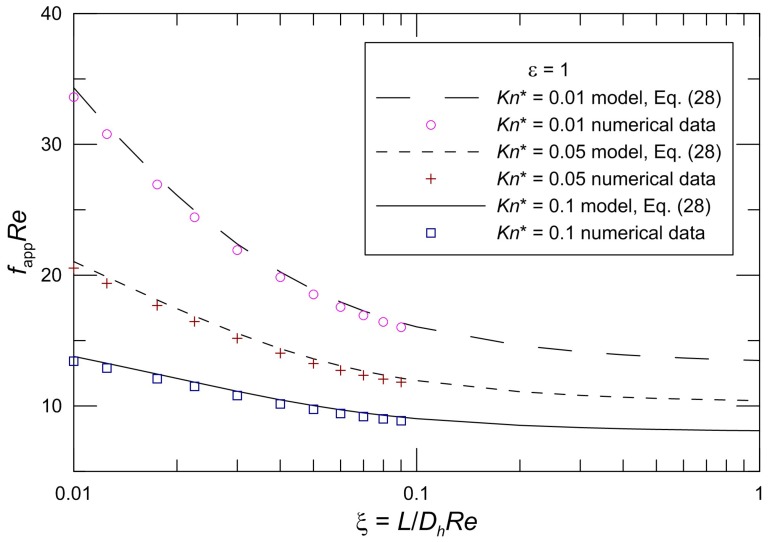
Comparison of *f*_app_*Re* for our numerical data.

Figure 6 demonstrates the corresponding varying local *f*_app_*Re* with the non-dimensional flow distance and the comparison between the proposed model Equation (28) and slip flow numerical results when the aspect ratio *ε* = 1. It is found that the model predictions agree with our numerical results. The maximum deviation between the numerical results and the proposed model is less than 2.6%. 

It is clear that Equation (28) characterizes the pressure drop in microchannel plate fin heat sinks. The maximum deviation of exact values is less than 8 percent. The pressure drop may be predicted from Equation (28), unless greater accuracy is desired. The developed pressure drop model may be suitable for the parametric design of and optimization studies on microchannel plate fin heat sinks.

The fluid flow behavior in the developing region differs from that in the fully developed region. The parameter $L/\left(D_{h}Re\right)$ is always a significant parameter in internal fluid flows. The flow behaves differently and is dominated by different mechanisms as the parameter $L/\left(D_{h}Re\right)$ changes. This effect of the developing region is significant if the microchannels are short. It is shown that the data presented by some researchers may display entrance effects. 

It can be seen that fully developed flow is attained at different *ξ* values, with low-aspect-ratio ducts reaching it a little earlier. The fully developed flow is attained when *ξ* is approximately 1 for rectangular ducts, as seen from these figures. The dimensionless developing length *ξ* is the proper parameter to take into account the entrance effects on the overall friction factor and pressure drop. The friction factor is higher in the developing flow region. Finally, beyond the entry region, the conventional theory for fully developed flow applies. Generally, the entrance region effects are less than 2% for the friction factor and Reynolds number product and can be neglected when $L/\left(D_{h}Re\right)\geq 1$. The fully developed flow (or long duct) criterion for pressure drop is $L/\left(D_{h}Re\right)\geq 1$.

## 5. Conclusions

This paper investigated pressure drop in the fluid flow in microchannel plate fin heat sinks. The paper deals with issues of hydrodynamic flow development in microchannel plate fin heat sinks. A model was proposed for predicting the pressure drop in microchannel plate fin heat sinks for developing slip flow and continuum flow. The accuracy of the developed model was found to be within 5 percent for practical configurations. Errors of this magnitude are acceptable for most engineering purposes. The model can be considered adequate and sufficiently reliable to analyze the pressure drop in microchannel plate fin heat sinks. As for slip flow, no solutions or tabulated data exist for microchannel plate fin heat sinks, so this developed model may be used to predict pressure drop in slip flow in microchannel plate fin heat sinks. The developed model is simple and founded on theory, and it may be used by the research community for the practical engineering design and optimization of microchannel plate fin heat sinks.

The goal of this investigation was to predict the pressure drop of the flow in microchannel plate fin heat sinks. The fully developed flow or long duct criterion for pressure drop is given. It is clearly shown that the entrance effects could be the source of the often-conflicting results previously reported in the literature.

## Nomenclature

*A*	flow area, m^2^
*a*	major semi-axis of rectangle, m
*b*	minor semi-axis of rectangle, m
*D_h_*	hydraulic diameter, = 4*A*/*P*
*f*	fanning friction factor, = $\bar{\tau }/\left(\frac{1}{2}\rho {\bar{u}}^{2}\right)$
*Kn*	Knudsen number, = $\lambda_{p}/D_{h}$
*Kn^*^*	modified Knudsen number, = *Kn*(2−*σ*)/*σ*
*L*	channel length, m
*P*	perimeter, m
*Po*	Poiseuille number, = $\bar{\tau }\;D_{h}/\mu \;\bar{u}$
*p*	pressure, $N\;/m^{2}$
*Re*	Reynolds number, = $\bar{u}D_{h}/\nu$
*T*	temperature, K
*t*	fin thickness, m
*U*	velocity scale, m/s
*u*	velocity, m/s
$\bar{u}$	average velocity, m/s
*X_n_*	function of *x*/*a*
*x, y*	Cartesian coordinates, m
*z*	coordinate in flow direction, m
Greek symbols	
*α*	constants
*γ*	ratio of specific heats
*δ_n_*	eigenvalues
*ε*	aspect ratio, = *b*/*a*
*λ*	hydrodynamic slip length, m
*λ_p_*	molecular mean free path, m
*μ*	dynamic viscosity, $N\cdot \;s/m^{2}$
*ν*	kinematic viscosity, m^2^/s
*ξ*	dimensionless hydrodynamic channel length, = $L/\left(D_{h}Re\right)$
*σ*	tangential momentum accommodation coefficient
$\bar{\tau }$	wall shear stress, $N\;/m^{2}$
Subscripts	
*D_h_*	based upon the hydraulic diameter
*ns*	no-slip

## References

[1] Kandlikar S., Garimella S., Li D., Colin S., King M.R. (2006). Heat Transfer and Fluid Flow in Minichannels and Microchannels.

[2] Wang S.X., Yin Y., Hu C.X., Rezai P. (2018). 3D integrated circuit cooling with microfluidics. Micromachines.

[3] Hajmohammadi M.R., Alipour P., Parsa H. (2018). Microfluidic effects on the heat transfer enhancement and optimal design of microchannels heat sinks. Int. J. Heat Mass Transf..

[4] Jing D.L., Song S.Y., Pan Y.L., Wang X.M. (2018). Size dependences of hydraulic resistance and heat transfer of fluid flow in elliptical microchannel heat sinks with boundary slip. Int. J. Heat Mass Transf..

[5] Bahiraei M., Heshmatian S. (2018). Thermal performance and second law characteristics of two new microchannel heat sinks operated with hybrid nanofluid containing grapheme-silver nanoparticles. Energy Convers. Manag..

[6] Khan A.A., Kim S.M., Kim K.-Y. (2016). Evaluation of various channel shapes of a microchannel heat sink. Int. J. Air-Cond. Refrig..

[7] Ansari D., Kim K.-Y. (2016). Double-layer microchannel heat sinks with transverse-flow configurations. Trans. ASME J. Electron. Packag..

[8] Al Siyabi I., Khanna S., Sundaram S., Mallick T. (2019). Experimental and numerical thermal analysis of multi-layered microchannel heat sink for concentrating photovoltaic application. Energies.

[9] Shen H., Zhang Y.C., Wang C.-C., Xie G.N. (2018). Comparative study for convective heat transfer of counter-flow wavy double-layer microchannel heat sinks in staggered arrangement. Appl. Therm. Eng..

[10] Ansari D., Kim K.-Y. (2017). Performance analysis of double-layer microchannel heat sinks under non-uniform heating conditions with random hotspots. Micromachines.

[11] Radwan A., Ookawara S., Mori S., Ahmed M. (2018). Uniform cooling for concentrator photovoltaic cells and electronic chips by forced convective boiling in 3D-printed monolithic double-layer microchannel heat sink. Energy Convers. Manag..

[12] Jing D.L., He L. (2018). Thermal characteristics of staggered double-layer microchannel heat sink. Entropy.

[13] Wang S.L., Li X.Y., Wang X.D., Lu G. (2018). Flow and heat transfer characteristics in double-layered microchannel heat sinks with porous fins. Int. Commun. Heat Mass Transf..

[14] Xia G.D., Liu R., Wang J., Du M. (2016). The characteristics of convective heat transfer in microchannel heat sinks using Al_2_O_3_ and TiO_2_ nanofluids. Int. Commun. Heat Mass Transf..

[15] Hassani S.M., Khoshvaght-Aliabadi M., Mazloumi S.H. (2018). Influence of chevron fin interruption on thermo-fluidic transport characteristics of nanofluid-cooled electronic heat sink. Chem. Eng. Sci..

[16] Zargartalebi M., Azaiez J. (2018). Heat transfer analysis of nanofluid based microchannel heat sink. Int. J. Heat Mass Transf..

[17] Duangthongsuk W., Wongwises S. (2018). A comparison of the thermal and hydraulic performances between miniature pin fin heat sink and microchannel heat sink with zigzag flow channel together with using nanofluids. Heat Mass Transf..

[18] Xu F., Wu H.Y. (2018). Experimental study of water flow and heat transfer in silicon micro-pin-fin heat sinks. Trans. ASME J. Heat Transf..

[19] Ansari D., Kim K.-Y. (2018). Hotspot thermal management using a microchannel-pinfin hybrid heat sink. Int. J. Therm. Sci..

[20] Vinoth R., Kumar D.S. (2018). Experimental investigation on heat transfer characteristics of an oblique finned microchannel heat sink with different channel cross sections. Heat Mass Transf..

[21] Adewumi O.O., Bello-Ochende T., Meyer J.P. (2013). Constructal design of combined microchannel and micro pin fins for electronic cooling. Int. J. Heat Mass Transf..

[22] Khan A.A., Kim S.M., Kim K.-Y. (2015). Performance analysis of a microchannel heat sink with various rib configurations. J. Thermophys. Heat Transf..

[23] Chai L., Wang L., Bai X. (2018). Thermohydraulic performance of microchannel heat sinks with triangular ribs on sidewalls–Part 1: Local fluid flow and heat transfer characteristics. Int. J. Heat Mass Transf..

[24] Chai L., Wang L., Bai X. (2019). Thermohydraulic performance of microchannel heat sinks with triangular ribs on sidewalls–Part 2: Average fluid flow and heat transfer characteristics. Int. J. Heat Mass Transf..

[25] Chai L., Xia G.D., Wang H.S. (2016). Laminar flow and heat transfer characteristics of interrupted microchannel heat sink with ribs in the transverse microchambers. Int. J. Therm. Sci..

[26] Zhai Y.L., Xia G.D., Li Z.H., Wang H. (2017). Experimental investigation and empirical correlations of single and laminar convective heat transfer in microchannel heat sinks. Exp. Therm. Fluid Sci..

[27] Ou J., Perot B., Rothstein J.P. (2004). Laminar drag reduction in microchannels using ultrahydrophobic surfaces. Phys. Fluids.

[28] Sbragaglia M., Prosperetti A. (2007). A note on the effective slip properties for microchannel flows with ultrahydrophobic surfaces. Phys. Fluids.

[29] Maynes D., Jeffs K., Woolford B., Webb B.W. (2007). Laminar flow in a microchannel with hydrophobic surface patterned microribs oriented parallel to the flow direction. Phys. Fluids.

[30] Ybert C., Barentin C., Cottin-Bizonne C., Joseph P., Bocquet L. (2007). Achieving large slip with superhydrophobic surfaces: Scaling laws for generic geometries. Phys. Fluids.

[31] Ng C.-O., Wang C.Y. (2009). Stokes shear flow over a grating: Implications for superhydrophobic slip. Phys. Fluids.

[32] Feuillebois F., Bazant M.Z., Vinogradova O.I. (2009). Effective slip over superhydrophobic surfaces in thin channels. Phys. Rev. Lett..

[33] Zhang J.X., Yao Z.H., Hao P.F. (2016). Drag reductions and the air-water interface stability of superhydrophobic surfaces in rectangular channel flow. Phys. Rev. E.

[34] Lee C., Choi C.-H., Kim C.-J. (2016). Superhydrophobic drag reduction in laminar flows: A critical review. Exp. Fluids.

[35] Patlazhan S., Vagner S. (2017). Apparent slip of shear thinning fluid in a microchannel with a superhydrophobic wall. Phys. Rev. E.

[36] Antuono M., Durante D. (2018). Analytic solutions for unsteady flows over a superhydrophobic surface. Appl. Math. Model..

[37] Rastegari A., Akhavan R. (2018). The common mechanism of turbulent skin-friction drag reduction with superhydrophobic longitudinal microgrooves and riblets. J. Fluid Mech..

[38] Morini G.L., Spiga M. (1998). Slip flow in rectangular microtubes. Microscale Thermophys. Eng..

[39] Morini G.L. (2006). Scaling effects for liquid flows in microchannels. Heat Transf. Eng..

[40] Wang C.Y. (2013). Benchmark solutions for slip flow and H1 heat transfer in rectangular and equilateral triangular ducts. Trans. ASME J. Heat Transf..

[41] Wang C.Y. (2014). On the Nusselt number for H_2_ heat transfer in rectangular ducts of large aspect ratios. Trans. ASME J. Heat Transf..

[42] Steinke M.E., Kandlikar S.G. (2004). Review of single-phase heat transfer enhancement techniques for application in microchannels, minichannels and microdevices. Int. J. Heat Technol..

[43] Steinke M.E., Kandlikar S.G. (2006). Single-phase liquid friction factors in microchannels. Int. J. Therm. Sci..

[44] Ranjith S.K., Patnaik B.S.V., Vedantam S. (2013). Hydrodynamics of the developing region in hydrophobic microchannels: A dissipative particle dynamics study. Phys. Rev. E.

[45] Duan Z.P., Muzychka Y.S. (2010). Slip flow in the hydrodynamic entrance region of circular and noncircular microchannels. Trans. ASME J. Fluids Eng..

[46] Mishan Y., Mosyak A., Pogrebnyak E., Hetsroni G. (2007). Effect of developing flow and thermal regime on momentum and heat transfer in micro-scale heat sink. Int. J. Heat Mass Transf..

[47] Kohl M.J., Abdel-Khalik S.I., Jeter S.M., Sadowski D.I. (2005). An experimental investigation of microchannel flow with internal pressure measurements. Int. J. Heat Mass Transf..

[48] Bayraktar T., Pidugu S.B. (2006). Characterization of liquid flows in microfluidic systems. Int. J. Heat Fluid Flow.

[49] Barber R.W., Emerson D.R. A numerical investigation of low Reynolds number gaseous slip flow at the entrance of circular and parallel plate microchannels. Proceedings of the ECCOMAS Computational Fluid Dynamics Conference.

[50] Vocale P., Spiga M. (2013). Slip flow in the hydrodynamic entrance region of microchannels. Int. J. Microscale Nanoscale Therm. Fluid Transp. Phenom..

[51] Hettiarachchi H.D.M., Golubovic M., Worek W.M., Minkowycz W.J. (2008). Three-dimensional laminar slip-flow and heat transfer in a rectangular microchannel with constant wall temperature. Int. J. Heat Mass Transf..

[52] Sparrow E.M., Lundgren T.S., Lin S.H. (1962). Slip flow in the entrance region of a parallel plate channel. Proceedings of the Heat Transfer and Fluid Mechanics Institute.

[53] Quarmby A. (1965). Slip flow in the hydrodynamic entrance region of a tube and parallel plate channel. Appl. Sci. Rev..

[54] Ma N.Y., Duan Z.P., Ma H., Su L.B., Liang P., Ning X.R., Zhang X. (2018). Lattice Boltzmann simulation of the hydrodynamic entrance region of rectangular microchannels in the slip regime. Micromachines.

[55] Ebert W.A., Sparrow E.M. (1965). Slip flow in Rectangular and Annular Ducts. J. Basic Eng..

[56] Shah R.K., London A.L. (1978). Laminar Flow Forced Convection in Ducts.

[57] Duan Z.P., Muzychka Y.S. (2007). Slip flow in non-circular microchannels. Microfluid. Nanofluid..

[58] Kays W.M., London A.L. (1984). Compact Heat Exchangers.

[59] Curr R.M., Sharma D., Tatchell D.G. (1972). Numerical predictions of some three dimensional boundary layers in ducts. Comput. Methods Appl. Eng..

